# Cone-Beam Computed Tomographic Evaluation of Periapical Lesion Healing After Root Canal Preparation with Different File Systems

**DOI:** 10.3390/bioengineering12111267

**Published:** 2025-11-19

**Authors:** Alaa-Eldeen O. Mais, Amr M. Abdallah, Essam Osman, Hatem A. Alhadainy

**Affiliations:** 1 Division of Endodontics, Department of Restorative Sciences, Faculty of Dentistry, Beirut Arab University, Beirut, Beyrouth P.O. Box 11, Lebanon; alaa_mais@hotmail.com; 2 Division of Endodontics, Department of Restorative Dentistry, Faculty of Dentistry, Alexandria University, Alexandria 21526, Egypt; dr_amr_abdallah@hotmail.com; 3 Dental Biomaterials, Faculty of Dentistry, Alexandria University, Alexandria 21526, Egypt; essamosman11@gmail.com; 4 Division of Endodontics, Department of Dentistry, University of North Carolina, Chapel Hill, NC 27514, USA

**Keywords:** biomedical imaging, computer-aided assessment, cone-beam computed tomography, periapical radiolucency, nickel-titanium files, single file system, tornado rotary system

## Abstract

**Background**: Cone-beam computed tomography (CBCT) was used for a 1-year follow-up of a randomized clinical trial to compare a stainless-steel Tornado file system with OneShape and WaveOne rotary systems for biomechanical canal preparation, as indicated by radiolucency sizes of periapical lesions. **Methods**: Lower molars with necrotic pulps and periapical lesions were randomly divided into three groups (n = 20) according to three rotary file systems. After root canal treatment, clinical and assessment of the CBCT periapical index scores were blindly evaluated at one year using pre- and post-instrumentation CBCT images. Statistical analysis was performed to compare the three systems at a *p*-value of 0.05. **Results**: The results revealed a significant decrease in the size of apical radiolucency in each group after one-year follow-up, with no statistically significant difference among the three systems (*p* > 0.05). **Conclusions**: CBCT is a valuable biomedical imaging modality for assessing periapical lesion healing. Tornado, WaveOne, and OneShape systems can be used with similar efficacy for root canal preparation in teeth with periapical lesions. **Clinical Trial Registration**: The study was retrospectively registered with ClinicalTrials.gov (NCT06752837). Date of Registration: 30 December 2024. The CONSORT group has identified it as essential.

## 1. Introduction

The most accepted treatment for periapical lesions is nonsurgical root canal therapy (RCT), as effective root canal debridement followed by proper filling creates a favorable biological environment for healing. The chemo-mechanical preparation entails the elimination of microbial biofilms and infected soft and hard tissues [1]. Stainless-steel (SS) or nickel-titanium (Ni-Ti) instruments are used with irrigating solutions to achieve this goal [2]. Modern preparation techniques, employing rotary or reciprocating equipment, may not plan root canal walls, leaving behind un-instrumented dentin surfaces with residual tissue and microbial biomass, which may lead to persistent infection [3,4]. Effective chemo-mechanical preparation is directly related to the healing of periapical lesions, as it eliminates current infections and guards against future infections [5,6].

Several single-file systems have been promoted for shaping most canals, irrespective of their length, diameter, or curvature, such as WaveOne file (Dentsply Maillefer, Ballaigues, Switzerland). It is made of a unique Ni-Ti alloy known as M-Wire, which has resistance to cycle fatigue and enhanced instrument flexibility [7,8]. WaveOne files produce a reciprocal motion that calls for specialized automated equipment [9]. OneShape (Micro Mége, Besançon, France) is another single-file system, made of a standard austenite-Ni-Ti alloy with a single constant taper of 0.06 mm, a tip size of 0.25 mm, and distinct cross-sectional designs along working length. OneShape files are used in a clockwise rotation in a specialized automated handpiece [10].

The advantage of Ni-Ti instruments is their super elasticity, whereas their disadvantage is unexpected fatigue fracture [3]. The Tornado rotary system (MIB, France) was developed to address some Ni-Ti limitations. It is a rotating system made of SS, a passive inactive tip, and a constant taper of 0.04 mm [5]. The difference between these instruments and rotary Ni-Ti instruments is that they scrape the dentinal walls instead of cutting into dentin [11]. The Tornado system contains a brush called the “Finisher Brush” with six SS strands that extend automatically outward when used in a handpiece at 6500 rpm. Since irrigation of the root canal system with a needle and syringe does not produce enough hydrodynamic shear stresses to loosen the tissue or biofilms that stick to the root canal walls [6], agitation techniques have been introduced, provided by the Finisher Brush Tornado system, to mitigate this drawback [12].

The success of root canal treatment (RCT) has traditionally been evaluated through a combination of clinical examination and periapical radiographic assessment [13,14]. While these conventional methods remain fundamental, recent advances in biomedical imaging have markedly enhanced the precision and reproducibility of endodontic outcome assessment. Cone-beam computed tomography (CBCT) has emerged as a transformative imaging modality, providing high-resolution, three-dimensional (3D) visualization that enables detailed evaluation of periapical bone healing and volumetric changes in lesions. In parallel, the incorporation of automated and semi-automated image analysis algorithms has facilitated standardized quantification of lesion dynamics, thereby reducing observer bias and improving the consistency of longitudinal evaluations. Collectively, these technological innovations have strengthened the evaluative framework in endodontic research for comparing the biological performance of various root canal instrumentation systems in advancing evidence-based endodontic practice [15].

Despite the multifactorial nature of periapical lesion healing, substantial evidence indicates that successful outcomes are achieved in germ-free conditions and are strongly dependent on the thoroughness of root canal preparation [5,6]. While several investigations have evaluated and compared the cleaning and shaping efficacy of WaveOne and OneShape file systems, the impact of Tornado files on periapical tissue healing remains unexplored. This knowledge gap underscores the need for further research to elucidate the biological outcomes associated with different file system designs and kinematics in endodontic therapy. Therefore, healing the periapical lesions may reflect the efficiency of file systems used for canal preparation. We used CBCT to evaluate the efficiency of canal preparation with the Tornado rotary SS system compared with the WaveOne and OneShape rotary Ni-Ti single-file systems, controlling for other confounders. Comparing Tornado, WaveOne, and OneShape systems may provide clinically relevant evidence to guide the selection of instrumentation techniques that optimize periapical tissue repair and overall treatment outcomes. The null hypothesis states that periapical lesion healing does not differ significantly among teeth treated with Tornado, WaveOne, or OneShape file systems.

## 2. Materials and Methods

### 2.1. Registration and Ethical Approval

**Figure 1:**
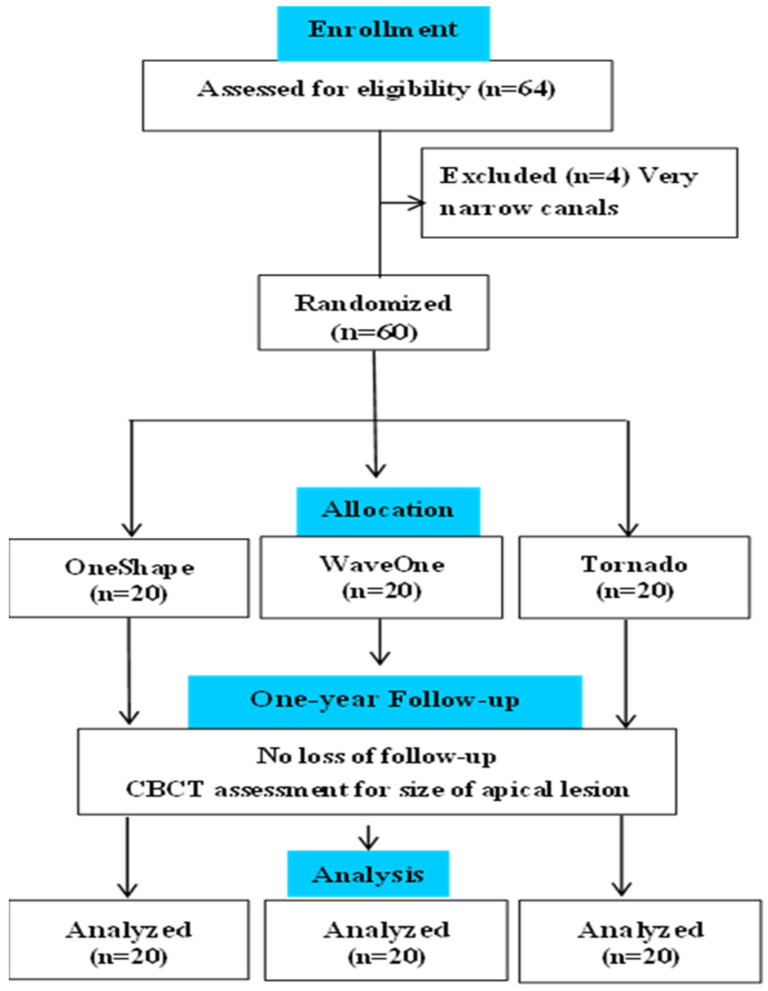
Consort Flow Diagram.

This study has been written according to the guidelines of the Preferred Reporting Items for Randomized Trials in Endodontics PRIRATE 2020 [16]. The protocol was registered as ClinicalTrials.gov (NCT06752837), and the date of Registration is 30 December 2024. The CONSORT Flow Diagram is presented in Figure 1. The protocol for this randomized clinical study received ethical clearance from the Institutional Review Board (IRB) in accordance with the Declaration of Helsinki, under approval code 2017-H-0056-D-P-0457.

### 2.2. Selection of Patients

Patients allocated to the Endodontics department for nonsurgical RCT were the study population. A sample size of 60 subjects (20 per group; 38 females and 22 males) was selected to provide 80% power at a two-sided significance level of 0.05 to detect a large group effect. This corresponds to a Cohen’s f ≈ of 0.40 for a one-way ANOVA with three equal groups (which approximates a pairwise Cohen’s d ≈ of 0.80). With these assumptions, 20 cases per group are sufficient to detect clinically meaningful differences in the CBCT PAI scores across groups. To be included in the study, patients should have lower molars with two separate mesial canals, distinct apical foramina, mature apices, within similar coronal defects, and neither cracks nor resorption. Canal curvature for mesial canals ranged from 15° to 45°. The study excluded patients younger than 16 years or older than 65, patients with diabetes, those with immune-compromising conditions, or those who had previous dental work on the working tooth. The inclusion/exclusion criteria were based on confounders justification for canal morphology, systemic health, and infection status, ensuring homogeneity across groups. We informed the patients about the potential risks, discomfort, and potential benefits. Patients who met the inclusion criteria and agreed to be included signed a consent form. Table 1 shows the demographics of the study samples.

### 2.3. Study Groups

Every patient was evaluated and reviewed through appropriate history-taking, clinical examination, and preoperative digital radiography. The past dental and medical history, major complaints, and demographic data were recorded. Operating teeth were scanned with an axial slice thickness of 0.1 mm using a CBCT (Kodak 9000C, Carestream, NY, USA) with an 80 kV, 4 mA, 51 × 51 mm field of view, and 0.1/voxel (mm) size. After the computer’s recording, patients were randomly divided into three equal groups (n = 20) using allocation concealment, sealed, opaque envelopes prepared by an independent assistant to keep the assignment sequence hidden from researchers and participants.

Group 1: The instrumentation was performed using a revolving SS Tornado rotary system (MIB, FRANCE).

Group 2: Instrumentation was performed using a reciprocating Ni-Ti WaveOne system (Dentsply Maillefer, Ballaigues, Switzerland).

Group 3: The instrumentation was performed using a rotative Ni-Ti OneShape system (Micro Mége, Besançon, France).

### 2.4. Root Canal Therapy

All procedures were performed by a single operator (AOM) to maintain standardization in operator skill. After proper anesthesia and isolation, access cavities were completed with a round bur, followed by an Endo Z bur. Working length was determined using an apex locator (DENTA PORT ZX, Kyoto, Japan), and confirmed by periapical X-ray. Canal patency was established with a #15 K-type file (MANI, Thái Nguyên, Vietnam) and RC prep glide path (Premier Dental, West Grove, PA, USA). Canals were prepared with the assigned instrumentation system according to the manufacturer’s instructions and irrigated with 2.0 mL of a 2.5% sodium hypochlorite solution (NaOCl) (Clorox, Beirut, Lebanon) followed by 3.0 mL of 17% ethylene-diamine-tetra-acetic acid (EDTA) (NEXABIO, Cheongju, Chungbuk, Republic of Korea) for 1 min, and then 1.3% NaOCl as final irrigation. Tornado Finisher was used as directed by the manufacturer at 1 mm shorter than the working length in an up-and-down motion. In the other two groups, irrigation was performed with a 31-G side-vented needle (Ultradent Products Inc., South Jordan, UT, USA) placed passively into the canal, 1 mm short of the working length.

Intracanal calcium hydroxide was used for canal filling for one week. Every tooth in the three groups underwent the same obturation procedure (Lateral condensation technique) using gutta-percha (DiaDent, Cheongju, Chungbuk, Republic of Korea) and a resin sealer (META BIOMED, Colmar, PA, USA). Finally, coronal preparations of all teeth were restored with composite filling (Ivoclar, Schaan, Switzerland).

### 2.5. Evaluation of Apical Healing

**Figure 2:**
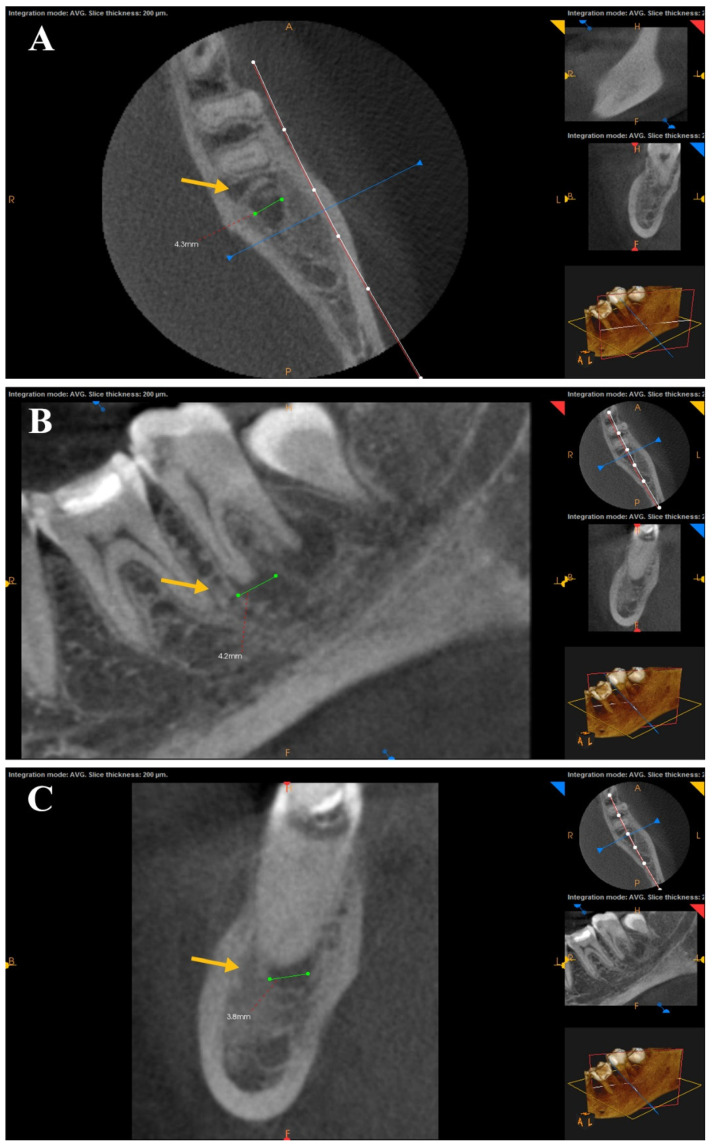
CBCT scans show the axial (**A**), sagittal (**B**), and coronal (**C**) planes. The largest extension of the lesion (yellow arrow) determined the score in the CBCT PAI.

The clinical evaluation was conducted at a one-year follow-up visit for each patient, including spontaneous pain, sinus tract, swelling, mobility, periodontal probing depths greater than baseline, or sensitivity to percussion or palpation [17]. A post-operative CBCT image was obtained and compared to the pre-operative one to assess the impact of canal preparation of each file on the periapical lesion based on Estrela et al. [18] CBCT periapical index (CBCT PAI) to obtain three orthogonal linear measurements (bucco-lingual, mesio-distal, and diagonal) for each lesion (Figure 2). The largest measured diameter was used for scoring with the CBCT-PAI. Two independent examiners performed measurements on the digital CBCT images using the software measurement calipers; the measurements were blinded concerning group allocation. Discrepancies were resolved by repeat measurement and consensus resolution (ICC = 0.91). This semi-automated, computer-aided approach improved measurement reproducibility and minimized observer bias. Cortical bone expansion (E) and cortical bone destruction (D) were included in the scoring system as appropriate [17] (Table 2). If one of these conditions was found in the CBCT analysis, the variables E and D were added to each score.

In addition to within-group comparisons, we computed a change score for each tooth (pre-treatment CBCT-PAI score minus 1-year CBCT-PAI score) and compared these change scores across groups using the Kruskal–Wallis test (non-parametric, ordinal data). Where appropriate, pairwise comparisons were performed using Mann–Whitney U tests with Bonferroni correction.

### 2.6. Analytical Statistics

A qualitative analysis of clinical evaluations was conducted for signs and symptoms. The pre- and post-operative Estrela et al. index scores were recorded. The Statistical Package for the Social Sciences software (SPSS 22, SPSS Inc., Chicago, IL, USA) was used for statistical analysis. Frequency was used to describe group qualitative data, and normal distribution was tested using the Shapiro–Wilk test. Since the data did not follow a normal distribution, the scores of lesion size before and after one year were collected for each group using the Wilcoxon signed-rank test, while the inter-group comparison was performed using the Kruskal–Wallis test followed by the Mann–Whitney test for pair-wise comparison. All statistical tests were performed at a *p*-value of 0.05.

In addition to within-group comparisons, we computed a change score for each tooth (pre-treatment CBCT-PAI score minus 1-year CBCT-PAI score) and compared these change scores across groups using the Kruskal–Wallis test (non-parametric, ordinal data). Where appropriate, pairwise comparisons were performed using Mann–Whitney U tests with Bonferroni correction.

## 3. Results

The participating patients were 38 females (63.3%) and 22 males (36.7%), with a mean age of 35.7 ± 10.1, ranging from 16 to 65 years. The inter-group statistical analysis of baseline confounder variables (gender, age, angle of canal curvature, and tooth type) was performed using one-way ANOVA and chi-square tests, confirming no significant differences between groups (*p* > 0.05).

After a year of follow-up, all patients showed no clinical symptoms. There was no pain, sinus tract, swelling, mobility, sensitivity to percussion, or periodontal probing depths greater than baseline. Frequencies of the CBCT PAI scores are presented in Table 3 for the apical lesions of all groups before and after one year. Healing scores after one year revealed that OneShape showed the highest frequency of 0 score in 10 cases, followed by Tornado in 6 cases. WaveOne showed 4 cases with a “score 1”, while Tornado showed two cases. “Score 2” of the healing assessment was the most prominent as it appeared in 12 cases of Tornado, 14 cases of WaveOne, and 8 cases of OneShape. “Score 3” appeared in two cases of both WaveOne and OneShape, and no case showed “score 4” or “score 5”.

**Figure 3:**
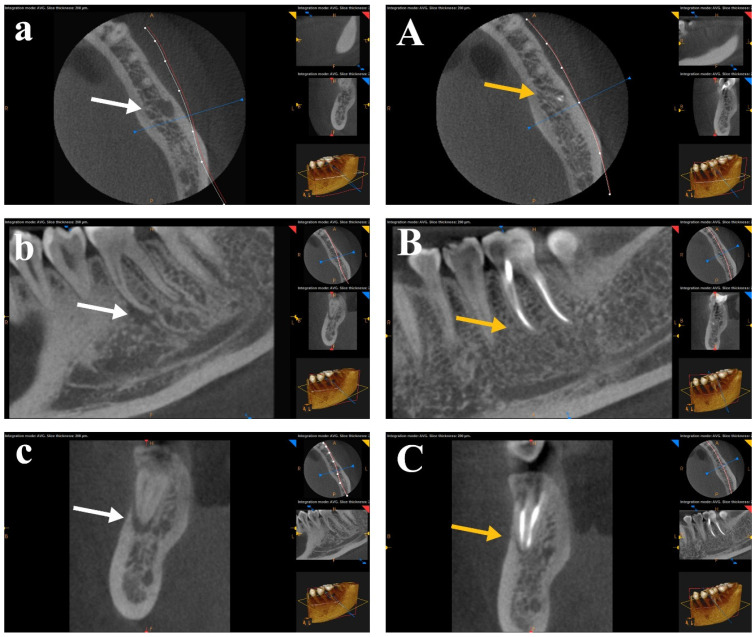
CBCT scans of the Tornado group before (**a**–**c**) and after one year (**A**–**C**). Complete healing was detected in the axial, sagittal, and coronal views (arrows, the white is “before” and the yellow is “after” treatment).

**Figure 4:**
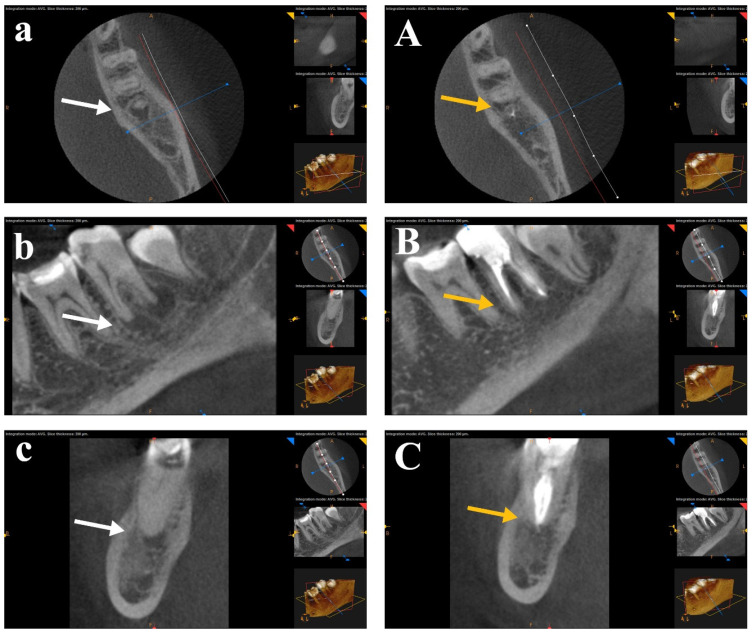
CBCT scans of the WaveOne file group before (**a**–**c**) and after one year (**A**–**C**). Complete healing was detected in the axial, sagittal, and coronal views (arrows, the white is “before” and the yellow is “after” treatment).

**Figure 5:**
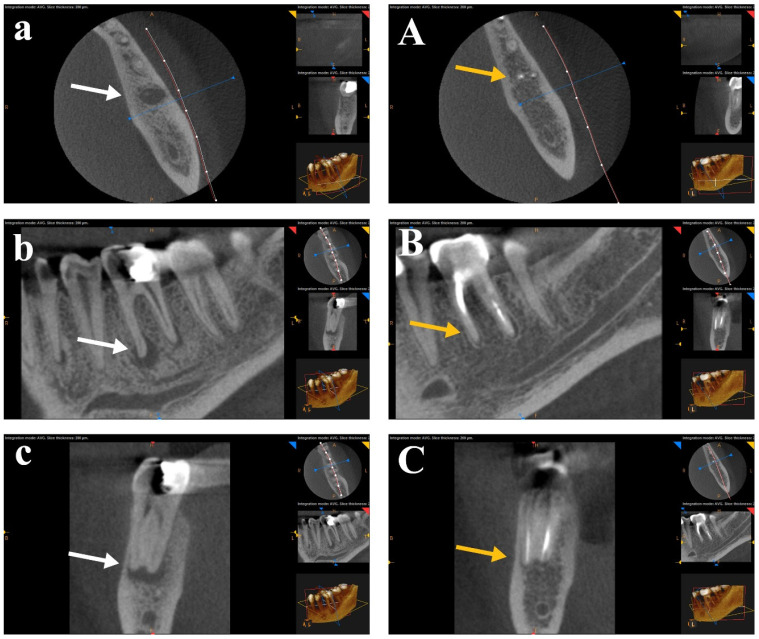
CBCT scans of the OneShape file group before (**a**–**c**) and after one year (**A**–**C**). Complete healing was detected in the axial, sagittal, and coronal views (arrows, the white is “before” and the yellow is “after” treatment).

A statistical comparison of preoperative periapical lesions was performed using CBCT images across the three study groups before root canal treatment. There was no significant difference between the tested groups in pre-operative CBCT images. The periapical lesions were statistically compared for each group before RCT and after one year. A Wilcoxon Signed-Rank Test showed a statistically significant difference (*p* < 0.05) for the mean ranks before RCT and after one year for each group. CBCT scans for each group showed healing of the periapical lesions after one year of follow-up. No statistical significance was found in post-operative CBCT images between the three tested groups. The difference between the healing of periapical lesions after canal preparation with the rotary SS Tornado system was nonsignificant compared to WaveOne and OneShape rotary Ni-Ti single-file systems. Figure 3, Figure 4 and Figure 5 show CBCT images of preapical lesions before root canal preparation and after one year for the three groups. Complete healing was detected in the axial, sagittal, and coronal views of the three file systems.

The scores changes indicated lesion reduction in all groups; however, the between-group comparison using the Kruskal–Wallis test did not reach statistical significance (*p* > 0.05), and no pairwise comparison between Tornado, WaveOne, and OneShape reached significance after Bonferroni correction. These results are consistent with the per-group Wilcoxon signed-rank tests showing within-group improvement but no demonstrable superiority of any single system in the magnitude of score change at one year.

## 4. Discussion

CBCT, as a biomedical imaging and computer-aided tool, enables precise, 3D visualization of periapical structures and dynamic monitoring of lesion resolution over time. Unlike conventional two-dimensional radiography, CBCT provides volumetric datasets that can be processed using computer-assisted algorithms to quantitatively evaluate bone density changes and lesion volume reduction with high reproducibility [19,20]. This digital assessment approach enhances the objectivity of endodontic outcome evaluation and supports evidence-based comparison between instrumentation systems. In the present study, CBCT imaging served as both a diagnostic and evaluative tool for detailed analysis of periapical healing patterns following root canal preparation with different file systems.

Ramis-Alario et al. [21] demonstrated that postoperative follow-up of periapical surgery using conventional 2D radiography can provide clinically reliable information when CBCT is unavailable. However, their findings revealed that periapical lesions measured using CBCT were significantly larger than those assessed with periapical or panoramic radiographs. Although CBCT remains a valuable modality for improving the accuracy and reproducibility of endodontic outcome evaluations, its limitations include radiation exposure, variability in image resolution, and limited accessibility, which must be acknowledged. In a complementary study, Musu et al. [22] highlighted the potential of ultrasound imaging as a non-ionizing and dynamic approach for assessing bone lesion healing. They reported that color power Doppler ultrasound may serve as a feasible tool for monitoring both early and long-term healing responses of intraosseous jaw lesions following surgical and nonsurgical treatment.

The healing of periapical lesions serves as a clinically meaningful and biologically relevant parameter for evaluating the efficiency of root canal preparation. Since periapical pathology is primarily caused by microbial infection within the root canal system, effective chemo-mechanical debridement is essential to create an environment conducive to healing. The extent to which an instrument can shape and clean the canal directly influences the elimination of residual bacteria and necrotic tissue, which are key factors in the resolution of periapical inflammation and bone regeneration (1,5). Unlike in vitro assessments of debris removal, evaluating lesion healing provides a long-term, outcome-based measure of successful canal preparation in clinical settings. Although periapical healing is multifactorial and influenced by systemic and procedural variables, the choice of instrumentation system plays a central role in ensuring adequate canal disinfection and shaping, thus justifying its use as a comparative clinical endpoint [6].

Although OneShape (n = 10) and Tornado (n = 6) produced more cases achieving a CBCT-PAI Score 0 than WaveOne (n = 0) at one year, this numerical imbalance may not reach statistical significance in between-group comparisons (Kruskal–Wallis on change scores; *p* > 0.05). Several non-exclusive explanations are possible: (1) random case allocation and residual heterogeneity in lesion chronicity or microanatomy that were not fully captured by the inclusion criteria; (2) modest differences in cleaning kinematics that may produce small effect sizes below our detection threshold; or (3) differences in the biological pace of bone remodeling as some systems’ treated teeth may require more time to show radiographic complete resolution. Given the absence of a statistically significant between-group effect and the small group sizes, we recommend cautious interpretation and call for larger, prospective studies stratified by lesion size/chronicity to explore whether any of these factors account for the observed numerical differences.

The Tornado group showed a significant decrease in the size of the apical lesion after one year in the Neelakantan et al. [11] study, when evaluating the effectiveness of the Tornado brush cleaning system compared to the syringe-and-needle irrigation technique. They found a significant variation in the amount of pulp tissue left in Ni-Ti groups compared to the Tornado group. They concluded that the debridement of canals prepared with the Tornado file system was enhanced by additional irrigating agitation using the Finisher Brush, which scrapes the canal walls to remove attached tissue and microbial biofilms. Garip et al. [23] reported that the high rotational speed of the Finisher Brush may produce centrifugal forces strong enough to push irrigating solution into the canal eccentricities, making the canals noticeably clean. The file edge is guided into the canal by its smooth, polished surface to remove the canal dentin symmetrically; it outperformed the Pro-Taper Universal system in terms of cleanliness [24].

WaveOne system significantly decreased the size of the apical lesion after one year compared to the initial lesion, which could explain the effectiveness of file cleaning. WaveOne file has a fixed taper of 0.08 mm for the first 4 mm and a special tapered design with a gradually decreasing percentage afterward. This tapering results in the removal of more debris in the coronal direction. The reverse helix of WaveOne helps to preserve and enhance flexibility in the coronal two-thirds of the completed preparation [25].

In the OneShape group, a significant decrease in the size of the apical lesion was observed after one year compared to the initial lesion. The distinctive design of OneShape facilitates a better upward removal of dentin debris, contributing to its good cleaning efficiency. The comparatively low tendency to retain debris may result from the large chip space, which pushes more debris in the coronal direction. It also boasts a variable helix angle that effectively removes upward debris and reduces the screwing effect [26].

WaveOne and OneShape outcomes may be attributed to the process of canal preparation with a single-file technique, which typically requires three repeated insertions. Multi-file systems are used multiple times in each canal, rubbing on its walls more frequently than a single file, to complete the preparation in a traditional full sequence technique. It was reported that as more files are inserted, more debris is created and compacted along the dentin walls, making it harder to flush out of the canal [26]. This explanation may be taken in favor of canal preparation with single-file systems.

No significant difference was found after one year when comparing the healing of periapical lesions using Tornado, WO, and OS for root canal preparation. In an earlier study, we assessed the shaping ability of the same three rotary systems, with pre- and post-instrumentation CBCT images. Our results in all tested levels showed no statistically significant difference in transportation and centering ability [27]. Based on these findings, we believe these file systems would be suitable for shaping root canals. The current study confirms previous findings on a practical scale and assesses the ability of these systems to provide high cleaning efficiency and periapical lesion healing ability.

To the best of our knowledge, no previous studies have directly compared the effect of Tornado files with WaveOne and OneShape systems on the healing of periapical lesions. However, numerous studies have compared WaveOne and OneShape’s cleaning effectiveness Al-Dulaimi et al. found that there was no discernible difference in the cleaning efficiency of the two files and that the OneShape, WaveOne, and Reciproc systems were significantly more effective than the ProTaper system in removing debris from root canals at all levels [26]. Another study by Azizi et al. [28] found that OneShape and WaveOne lower the bacterial load. However, Shaheen et al. [29] concluded that none of the single-file systems, WaveOne, OneShape, and XP-endo shaper, could achieve total canal cleanliness. Reciprocating systems generated more debris and smear layers than rotating instruments, as confirmed by Dagna et al. [30]. Ehsani et al. [31] and Sharma et al. [32] found that debris was extruded through the apex when WaveOne and OneShape were used in single canal premolars.

Given that periapical lesion healing is a multifactorial biological process influenced by both host and procedural factors, we investigated the association between endodontic file system performance and periapical lesion healing. Potential confounding variables were controlled by rigorous inclusion and exclusion criteria, ensuring that observed differences could be attributed primarily to the file system rather than to patient- or treatment-related factors. Although we found no statistically significant difference between the three file systems, clinical impact indicates that the SS rotary system can be used with the same efficiency as the NiTi system. Different outcomes may be attributed to limitations in clinical studies and to uncontrollable physiological variables. The grouping of patients in the study was not completely controlled, and other factors as immune profiles, canal morphology, and patient-specific healing responses, may have played a role in the outcomes.

Ethical approval for the study was obtained on 4 December 2017. Participant recruitment commenced on 12 July 2018, and the clinical trial registration was completed on 30 December 2024. The retrospective registration on ClinicalTrials.gov was due to the lack of institutional infrastructure and administrative support for trial registration at the time of study design. The study protocol and statistical analysis plan were finalized and ethically approved before participant enrollment began, ensuring that the late registration did not compromise the scientific integrity, methodological transparency, validity, or reliability of the results. The research team has established standardized procedures to guarantee that all future clinical trials are prospectively registered in full compliance with the International Committee of Medical Journal Editors and World Health Organization guidelines. Future studies with larger sample sizes and longer follow-up are recommended to investigate the healing of the periapical lesions after RCT with different file systems.

## 5. Conclusions

Within the limitations of this study, the integration of CBCT-based evaluation enabled precise 3D assessment of lesion reduction and bone regeneration. The results indicated that the stainless-steel Tornado rotary system exhibited healing trends comparable to those observed with the NiTi WaveOne and OneShape single-file systems. Overall, these findings highlight the potential of CBCT-supported analysis to advance evidence-based evaluation of root canal instrumentation systems and periapical tissue healing.

## Figures and Tables

**Table 1:** Demographics of the study samples.

Item	Data	
**Gender**	Male	22 (36.7%)
	Female	38 (63.3%)
**Age**	Mean	31.66667 years
	SD	7.067363
**Angle of curvature**	Mean	30.75°
	SD	6.832825
**Type of tooth**	46	20 (33.3%)
	47	10 (16.7%)
	36	18 (30%)
	37	12 (20%)
**Number of canals instrumented per tooth**	3	
**Number of cases assessed per group**	20	

**Table 2:** Periapical index.

Score	Quantitative Bone Alterations in Mineral
0	Intact periapical bone structures
1	Diameter of periapical radiolucency: 0.5–1 mm
2	Diameter of periapical radiolucency: 1–2 mm
3	Diameter of periapical radiolucency: 2–4 mm
4	Diameter of periapical radiolucency: 4–8 mm
5	Diameter of periapical radiolucency: 8 mm
Score (n) + E *	Expansion of periapical cortical bone
Score (n) + D *	Destruction of periapical cortical bone

* The variables cortical bone expansion (E) and cortical bone destruction (D) were added to each score if either of these conditions.

**Table 3:** Frequency of CBCTPAI scores for apical lesions of all groups before and after one year.

	Tornado		WaveOne		OneShape	
	Before	After	Before	After	Before	After
	Frequency					
0	0	6	0	0	0	10
1	0	2	0	4	0	0
2	4	12	0	14	0	8
3	6	0	10	2	16	2
4	10	0	10	0	4	0
5	0	0	0	0	0	0
Score (n) + E	0	0	0	0	0	0
Score (n) + D	0	0	0	0	0	0

## Data Availability

The original contributions presented in the study are included in the article/Supplementary Material, further inquiries can be directed to the corresponding author.

## Supplementary Materials

The following supporting information can be downloaded at: https://www.mdpi.com/article/10.3390/bioengineering12111267/s1, CONSORT 2010 checklist of information to include when reporting a randomized trial. This trial meets all essential CONSORT 2010 requirements for randomized clinical trial reporting [33].

## Abbreviations

The following abbreviations are used in this manuscript:

CBCT	Cone-beam computed tomography
RCT	Root canal therapy
SS	Stainless steel
Ni-Ti	Nickel-titanium
IRB	Institutional Review Board
PRIRATE	Preferred Reporting Items for Randomized Trials in Endodontics
NaOCl	Sodium hypochlorite solution
EDTA	Ethylene-diamine-tetraacetic acid
CBCT PAI	CBCT periapical index
SPSS	Social Sciences software
