# Supplementary material for: Cone-Beam Computed Tomographic Evaluation of Periapical Lesion Healing After Root Canal Preparation with Different File Systems
Source: Bioengineering (Basel). 2025 Nov 19;12(11):1267. doi: 10.3390/bioengineering12111267 (PMC12650241; doi:10.3390/bioengineering12111267)
Supplement: Supplementary file 1 [file bioengineering-12-01267-s001.zip › bioengineering-3992600-supplementary.pdf]

CONSORT 2010 checklist of information to include when reporting a randomised trial*

Manuscript title:

Cone-Beam Computed Tomographic Evaluation of Periapical Lesion Healing After Root Canal Preparation with Different File Systems.

Authors:

Alaa-Eldeen O. Mais, Amr M. Abdallah, Essam Osman, Hatem A. Alhadainy

Journal: *Bioengineering (Special Issue: Biomedical Imaging and Computer-Aided Assessment)*

Section/Topic	Item No.	Checklist Item (CONSORT 2010)	Reported in Manuscript (Section / Page)
**Title and Abstract**	1a	Identification as a randomized trial in the title	Title Page 1 "Randomized Clinical Trial"
	1b	Structured summary of trial design, methods, results, and conclusions	Abstract, p.1 – includes randomization, blinding, outcomes, and limitations
**Introduction**	2a	Scientific background and rationale	Introduction, pp. 1–3 – explains periapical lesion healing and rationale for file system comparison
	2b	Specific objectives or hypotheses	Introduction, p. 3 – includes null hypothesis on lesion healing equivalence among systems
**Methods – Trial Design**	3a	Description of trial design (e.g., parallel, allocation ratio)	Section 2.1 – Randomized, three-arm parallel trial, equal allocation (1:1:1)
	3b	Important changes to methods after trial commencement	Not applicable (no post-commencement changes)
**Participants**	4a	Eligibility criteria for participants	Section 2.2, pp. 3 – detailed inclusion/exclusion criteria (mature apices, canal curvature, systemic conditions)
	4b	Settings and locations where data were collected	Section 2.2 – Endodontics Department, Beirut Arab University
**Interventions**	5	Precise details of interventions for each group, including how and when they were administered	Section 2.4, pp. 4–5 – full procedural details for Tornado, WaveOne, and OneShape systems including irrigation protocols
**Outcomes**	6a	Clearly defined primary and secondary outcomes	Section 2.5, p. 5 – Primary: CBCT-PAI score change; Secondary: clinical signs (pain, swelling, mobility)
	6b	Any changes to trial outcomes after commencement	None reported
**Sample Size**	7a	How sample size was determined	Section 2.2, p. 3 – power 80%, α = 0.05, Cohen's f = 0.40 (large effect)
	7b	When applicable, explanation of interim analyses and stopping	Not applicable

		guidelines	
**Randomization – Sequence Generation**	8a	Method used to generate the random allocation sequence	Section 2.3, p. 4 – computer-generated randomization
	8b	Type of randomization; details of any restriction (e.g., blocking, stratification)	Simple randomization, equal group allocation (n = 20 per group)
**Allocation Concealment**	9	Mechanism used to implement the random allocation sequence (e.g., sealed envelopes)	Section 2.3, p. 4 – sealed opaque envelopes handled by an independent assistant
**Implementation**	10	Who generated the sequence, enrolled participants, and assigned interventions	Sequence generated by statistician; enrollment and treatment by a single operator (AOM); allocation by independent staff
**Blinding (Masking)**	11a	If done, who was blinded after assignment to interventions	Section 2.5, pp. 5–6 – CBCT examiners blinded to group allocation
	11b	Similarity of interventions if relevant	Comparable canal instrumentation approaches and irrigation volumes; Tornado's Finisher Brush noted as procedural difference
**Statistical Methods**	12a	Statistical methods used to compare groups for primary and secondary outcomes	Section 2.6, p. 6 – Wilcoxon, Kruskal–Wallis, Bonferroni correction, effect sizes
	12b	Methods for additional analyses, such as subgroup or adjusted analyses	None performed due to limited sample size
**Results – Participant Flow**	13a	Flow of participants through each stage (numbers randomly assigned, treated, analyzed)	Figure 1 – CONSORT Flow Diagram
	13b	Losses and exclusions after randomization, with reasons	None – all 60 patients completed the 1-year follow-up
**Recruitment**	14a	Dates defining periods of recruitment and follow-up	Discussion, p. 6 – recruitment: July 12, 2018 – July 2023; follow-up: 1 year
	14b	Why the trial ended or was stopped	Completed as planned after 1-year follow-up
**Baseline Data**	15	Baseline demographic and clinical characteristics for each group	Table 1 – demographic and canal morphology comparability (p > 0.05)
**Numbers Analyzed**	16	Number of participants analyzed per group	Results, p. 6 – all 60 cases (20 per group) analyzed
**Outcomes and Estimation**	17a	For each primary and secondary outcome, results with effect size and precision	Results, pp. 6–7 – includes medians, IQRs, effect sizes (r), p-values
	17b	For binary outcomes, present both absolute and relative effect sizes	Not applicable (ordinal data)
**Ancillary Analyses**	18	Subgroup or adjusted analyses	Not applicable

**Harms**	19	All important harms or unintended effects in each group	None observed
**Discussion**	20	Limitations of the trial, addressing potential sources of bias or imprecision	Discussion, pp. 10-12 – discusses operator bias, sample size, procedural confounders, retrospective registration
	21	Generalizability (external validity) of the trial findings	Discussion, p. 10 – cautions on extrapolation and need for larger studies
	22	Interpretation consistent with results, balancing benefits and harms	Conclusion, p. 11 – emphasizes comparability and methodological constraints
**Other Information**	23	Registration number and name of trial registry	Abstract and Section 2.1 – ClinicalTrials.gov NCT06752837
	24	Where the full trial protocol can be accessed	Not applicable (protocol included within manuscript methods)
	25	Sources of funding and role of funders	Funding statement – “No author received any funding.”

Summary

This trial meets all essential CONSORT 2010 requirements for randomized clinical trial reporting.

Items 3b, 6b, 7b, 12b, 17b, 18, and 24 are marked *not applicable* due to the exploratory single-center design and absence of protocol amendments or subgroup analyses.

Citation: Schulz KF, Altman DG, Moher D, for the CONSORT Group. CONSORT 2010 Statement: updated guidelines for reporting parallel group randomised trials. BMC Medicine. 2010;8:18. © 2010 Schulz et al. This is an Open Access article distributed under the terms of the Creative Commons Attribution License (http://creativecommons.org/licenses/by/2.0), which permits unrestricted use, distribution, and reproduction in any medium, provided the original work is properly cited.

*We strongly recommend reading this statement in conjunction with the CONSORT 2010 Explanation and Elaboration for important clarifications on all the items. If relevant, we also recommend reading CONSORT extensions for cluster randomised trials, non-inferiority and equivalence trials, non-pharmacological treatments, herbal interventions, and pragmatic trials. Additional extensions are forthcoming: for those and for up-to-date references relevant to this checklist, see www.consort-statement.org.
